# Comparison of the effects of radiotherapy doses of 50.4 Gy and 60 Gy on outcomes of chemoradiotherapy for thoracic esophageal cancer: subgroup analysis based on the Comprehensive Registry of Esophageal Cancer in Japan from 2009 to 2011 by the Japan Esophageal Society

**DOI:** 10.1007/s10388-019-00711-x

**Published:** 2020-01-07

**Authors:** Kenji Nemoto, Shohei Kawashiro, Yasushi Toh, Hodaka Numasaki, Yuji Tachimori, Takashi Uno, Keiichi Jingu, Hisahiro Matsubara

**Affiliations:** 1 Japan Esophageal Society, Tokyo, Japan; 2 Department of Radiology, Yamagata University Graduate School of Medicine, 2-2-2, Iida-nishi, Yamagata, 990-9585 Japan; 3 Department of Gastroenterological Surgery, National Hospital Organization Kyushu Cancer Center, Fukuoka, Japan; 4 Department of Functional Diagnostic Science, Osaka University Graduate School of Medicine, Osaka, Japan; 5 Center for Cancer Treatment, Kawasaki Saiwai Hospital, Kawasaki, Japan; 6 Department of Radiology, Chiba University Hospital, Chiba, Japan; 7 Department of Radiation Oncology, Tohoku University Graduate School of Medicine, Sendai, Japan; 8 Department of Frontier Surgery, Chiba University Graduate School of Medicine, Chiba, Japan

**Keywords:** Esophageal cancer, Concurrent chemoradiotherapy, Radiotherapy dose, Nationwide survey

## Abstract

**Background:**

In definitive chemoradiotherapy (CRTx) for esophageal cancer, a radiotherapy (RT) dose of 50.4 Gy in 28 fractions has been the standard in many countries, while 60 Gy in 30 fractions has been frequently used in Japan. To clarify the optimal RT dose in CRTx for esophageal cancer, we compared clinical outcomes with the two doses using data from the Comprehensive Registry of Esophageal Cancer in Japan by the Japan Esophageal Society (JES).

**Methods:**

Of the patients enrolled in the registry for 2015–2017 surveys (patients treated between 2009 and 2011), 996 patients who received definitive CRTx with 50.4 Gy or 60 Gy for thoracic esophageal cancer were eligible for analysis.

**Results:**

The complete response (CR) rates in the 50.4 Gy and 60 Gy groups were 49.1% and 46.4%, respectively (*p* = 0.5851). The 5-year overall survival (OS) rates in the 50.4 Gy group and 60 Gy group for stages I, II/III and IV were 64.2% and 57.2%, 35.0% and 27.0%, and 18.0% and 15.3%, respectively. Since no significant difference was found between the two groups, the 50.4 Gy group was not inferior to the 60 Gy group with regard to OS.

**Conclusions:**

The analysis revealed that the 50.4 Gy group had a non-inferior outcome in comparison with the 60 Gy group for stages I, II/III and IV thoracic esophageal cancer. These results were obtained from a large database for the first time in Japan.

## Introduction

Chemoradiotherapy (CRTx) has been the standard of care for patients with esophageal cancer who are not candidates for resection due to medically inoperable patients, unresectable locally advanced disease or refusal to undergo surgery. The efficacy of CRTx for esophageal cancer has been demonstrated in some previously conducted clinical trials [1–3]. In CRTx for esophageal cancer, a radiation therapy (RT) dose of 50.4 Gy in 28 fractions has been the global standard based on results of the INT 0123 phase III trial [4]. However, some radiation oncologists still insist that 50.4 Gy might be inadequate for definitive RT for esophageal cancer since some groups reported that a higher RT dose improved prognosis [5, 6]. In a patterns of care study in Japan, Kenjo et al. also showed that the median RT dose for esophageal cancer was 60 Gy in 30 fractions [7].

In this study, we compared the clinical outcomes of CRTx with RT doses of 50.4 Gy and 60 Gy using data from the Comprehensive Registry of Esophageal Cancer in Japan by the Japan Esophageal Society (JES) to clarify the optimal RT dose in CRTx for esophageal cancer.

## Materials and methods

### Patients

Among the patients enrolled between 2015 and 2017 (treated between 2009 and 2011) in the Comprehensive Registry of Esophageal Cancer in Japan by JES, we first extracted patients who received RT alone (RTx) or CRTx for esophageal cancer as curative intent. Only patients who were treated with external beam RT were included. Patients with cervical or abdominal esophageal cancer and pediatric patients were excluded. After exclusion, 388 and 1964 patients met the above eligibility criteria for RTx and CRTx, respectively (2352 patients in total) for the 3-year period mentioned above. The prognosis at 5 years after registry was also available for these patients. In this study, we analyzed data for patients who received definitive CRTx for esophageal cancer. Of those patients, data for patients who received an RT dose of 50.4 Gy or 60 Gy were analyzed.

### Data analysis

We assessed the correlations of RT doses with therapeutic efficacy (response) and prognosis. The Response Evaluation Criteria for Solid Tumors (RECIST) were used for analysis of the response [8]. We evaluated a stage of each patient’s disease based on the 7th edition of TNM classification by Union for International Cancer Control (UICC). Patients with missing data were excluded from the analysis. For statistical analyses, the Mann–Whitney test and *χ*^2^ test were used for continuous and categorical variables, respectively. The Kaplan–Meier method was used to estimate overall survival (OS). The log-rank test was used to compare OS rates in the two RT dose groups. SAS 9.4 (SAS Institute Inc., Cary, NC, USA) was used for statistical analysis. For all statistical analyses, *p* < 0.05 was considered statistically significant.

## Results

Data for 996 patients including 171 patients who received 50.4 Gy and 825 patients who received 60 Gy were used for analyses. The patient characteristics are shown in Table 1. There was no statistically significant difference in sex, histology, cN stage, or cStage between the two groups. In both groups, the tumor histology was squamous cell carcinoma in more than 90% of the cases. There were significant differences between the two groups in age, tumor location, cT stage and elective nodal irradiation. A larger proportion of patients in the 60 Gy group had a primary tumor at the middle thoracic esophagus, while the proportion of patients with a primary tumor at the lower thoracic esophagus was larger in the 50.4 Gy group (*p* = 0.0133). As for cT stage, the proportion of patients with cT1a, cT2, cT3 and cT4a was larger in the 50.4 Gy group, whereas the proportion of patients with cT1b and cT4b was larger in the 60 Gy group (*p* = 0.0001). The numbers of patients with T1aN0M0 and T1bN0M0 in cStage I were 7 (4.1%) and 15 (8.8%) in the 50.4 Gy group and 29 (3.5%) and 134 (16.2%) in the 60 Gy group, respectively.

**Table 1:** Patient characteristics

Characteristic	50.4 Gy (*n* = 171)		60 Gy (*n* = 825)		*p* value
	*n*	%	*n*	%	
Sex					
Male	147	86.0	731	88.6	0.3307
Female	24	14.0	94	11.4	
Age (years)					
Median (range)	68 (40–86)		69 (37–91)		0.006
Histology					
SCC	161	94.7	790	96.5	0.1298
AD	6	3.5	11	1.3	
Other	3	1.8	18	2.2	
Location					
Upper	38	22.2	177	21.5	0.0133
Middle	79	46.2	468	56.7	
Lower	54	31.6	180	21.8	
cT					
cTx	0	0.0	2	0.2	0.0001
cT0	0	0.0	0	0.0	
cT1a	14	8.2	34	4.1	
cT1b	27	15.8	173	21.0	
cT2	24	14.0	96	11.6	
cT3	65	38.0	242	29.3	
cT4a	24	14.0	83	10.1	
cT4b	17	9.9	195	23.6	
cN					
cNx	0	0.0	0	0.0	0.554
cN0	47	27.5	269	32.6	
cN1	63	36.8	274	33.2	
cN2	41	24.0	199	24.1	
cN3	20	11.7	83	10.1	
cStage					
cStage I	34	19.9	219	26.6	0.1493
cStage II–III	96	56.1	442	53.6	
cStage IV	41	24.0	164	19.9	
Elective nodal irradiation					
Yes	75	43.9	401	48.6	0.0011
No	72	42.1	375	45.5	
Unknown	24	14.0	49	5.9	

*SCC* squamous cell carcinoma, *AD* adenocarcinoma

Regarding the relationship between RT doses and response, the rates of complete response (CR) in the 50.4 Gy group and 60 Gy group were 49.1% and 46.4%, respectively (Table 2). As a comparison, the two groups showed that there was no statistically significant difference in the response rate (*p* = 0.5851).

**Table 2:** Relationship between dose and response

	50.4 Gy (*n* = 171)		60 Gy (*n* = 825)		*p* value
	*n*	%	*n*	%	
CR	84	49.1	383	46.4	0.5851
Non-CR/non-PD	73	42.7	354	42.9	
PD	14	8.2	88	10.7	

*CR* complete response, *PD* progressive disease

**Fig. 1:**
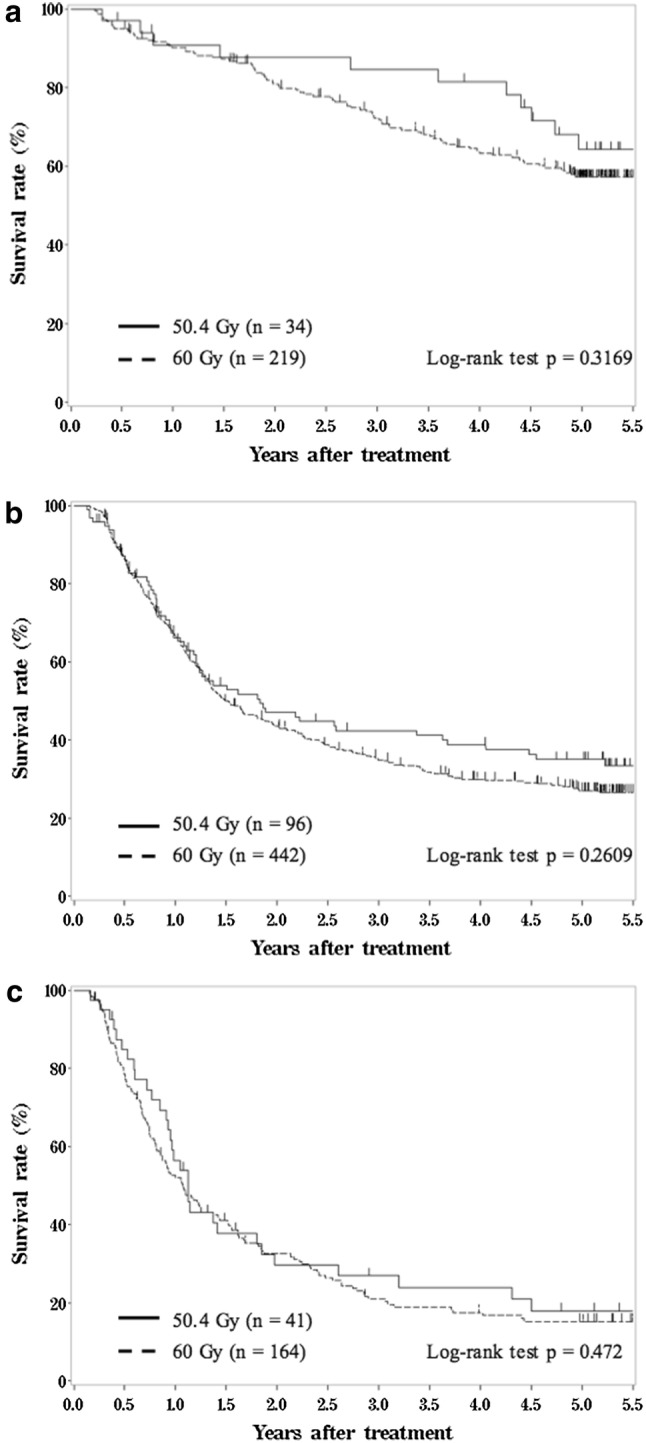
Kaplan–Meier estimates of overall survival for patients with stage I (**a**), stage II/III (**b**) and stage IV (**c**)

The Kaplan–Meier estimates of OS for clinical stages I, II/III and IV according to RT doses are shown in Fig. 1. The 5-year OS rates for stage I were 64.2% (95% confidence interval [CI] 44.5–78.5%) in the 50.4 Gy group and 57.2% (95% CI 49.9–63.8%) in the 60 Gy group (Fig. 1a). The 5-year OS rates for stage II/III were 35.0% (95% CI 25.2–45.0%) in the 50.4 Gy group and 27.0% (95% CI 22.7–31.5%) in the 60 Gy group (Fig. 1b). The 5-year OS rates for stage IV were 18.0% (95% CI 7.6–31.9%) in the 50.4 Gy group and 15.3% (95% CI 10.1–21.6%) in the 60 Gy group (Fig. 1c). Since a comparison of the two RT dose groups for OS showed no statistically significant difference, the 50.4 Gy group was not inferior to the 60 Gy group in all stages.

## Discussion

Our analyses showed that the outcome of CRTx with an RT dose of 50.4 Gy was not inferior to that of CRTx with a dose of 60 Gy for esophageal cancer, being similar to the results of the INT 0123 trial [4]. These results are shown for stages I, II/III and IV. It was difficult to clarify the factor affecting these results as was also the case in the INT 0123 trial. In our study, we did not divide the eligible patients into groups according to dose ranges, such as < 56 Gy, 56–66 Gy and > 66 Gy, but selected patients treated with two specific RT doses (50.4 Gy and 60 Gy) as we intended to exclude patients in whom CRTx was ceased due to the deterioration of their general condition and/or treatment-related toxicities. The reason for the selection of either RT dose in each case was unclear from the database used in our current study. Although a statistically significant difference in outcomes for patients in cT stage was observed between the two RT dose groups, it was difficult to clearly explain how the dose was selected for each patient. However, since the proportion of patients who received 60 Gy was larger than the proportion of patients who received 50.4 Gy for patients with cT4b disease, the RT dose of 60 Gy might have been selected with the expectation of better tumor control because salvage surgery after CRTx for patients with cT4b was regarded as difficult. According to the results of a patterns of care study on RT for esophageal cancer in Japan, Kenjo et al. also reported that the median RT dose was 60 Gy [7].

With regard to the reason why there was no statistically significant difference in OS between the two dose groups, it is possible that patients in the 50.4 Gy group had less treatment-related toxicities, which could not be confirmed precisely in the registry that we used for analysis. As another reason, there might have been a difference in the rate of salvage surgery between the two RT dose groups, namely more patients in the 50.4 Gy group might have been treated with salvage surgery for recurrence after CRTx. Some studies have shown that although salvage surgery frequently causes complications, some patients might benefit from salvage surgery and have a relatively good prognosis [9, 10]. A study on non-small-cell lung cancer also revealed that RT dose escalation did not necessarily achieve improvement in the prognosis [11]. On the other hand, some studies on prostate cancer showed that dose escalation seemed to be effective for better prognosis [8, 12]. Thus, there seems to be difference between RT dose and prognosis according to the types of malignancies.

We recognize that some limitations exist in this study. This study was conducted using data from the Comprehensive Registry of Esophageal Cancer in Japan, in which detailed information on patient characteristics and adverse events was not available. With regard to patient characteristics, some items had significant differences between the two groups (Table 1). This might be partly because the large sample size could have contributed to the statistical significance. To control the difference of characteristics between the two groups, another statistical method such as propensity score matching could be implemented. However, the items registered in the database were limited. Thus, if unmeasured confounding factors existed, the influence due to those factors could not be controlled after all. In addition, if propensity score matching could make only a small number of pairs, unmatched cases in the registry could not be utilized and lower the power of statistical test. For these reasons, we did not employ propensity score matching. Regarding the factors which could be related to OS such as performance status, organ function, intensity of chemotherapy, adverse events and treatment after recurrence, they were not available for the present analysis, because those items were not included in the registry. This should also be recognized as a major limitation. Even with these limitations, since this registry is the largest database for esophageal cancer in Japan, it can be expected that more institutions would use 50.4 Gy as the standard RT dose for esophageal cancer.

## Conclusions

Although a statistically significant difference was not found, analysis using the Comprehensive Registry of Esophageal Cancer in Japan revealed that the treatment outcome of an RT dose of 50.4 Gy in 28 fractions was not inferior to the treatment outcome with 60 Gy in 30 fractions for stages I, II/III and IV esophageal cancer. Although the conclusions obtained in the study were not yet final and further research is expected, these results were obtained by analysis of data from a large database for the first time in Japan.
